# Imprinted DNA methylation of the *H19* ICR is established and maintained in vivo in the absence of Kaiso

**DOI:** 10.1186/s13072-024-00544-8

**Published:** 2024-06-05

**Authors:** Hitomi Matsuzaki, Minami Kimura, Mizuki Morihashi, Keiji Tanimoto

**Affiliations:** 1 Institute of Life and Environmental Sciences, Life Science Center for Survival Dynamics, Tsukuba Advanced Research Alliance (TARA), University of Tsukuba, Tennoudai 1-1-1, Tsukuba, Ibaraki 305-8577 Japan; 2 Graduate School of Science and Technology, University of Tsukuba, Tsukuba, Ibaraki Japan

**Keywords:** Genomic imprinting, Imprinting control region, DNA methylation

## Abstract

**Background:**

Paternal allele-specific DNA methylation of the imprinting control region (*H19* ICR) controls genomic imprinting at the *Igf2/H19* locus. We previously demonstrated that the mouse *H19* ICR transgene acquires imprinted DNA methylation in preimplantation mouse embryos. This activity is also present in the endogenous *H19* ICR and protects it from genome-wide reprogramming after fertilization. We also identified a 118-bp sequence within the *H19* ICR that is responsible for post-fertilization imprinted methylation. Two mutations, one in the five RCTG motifs and the other a 36-bp deletion both in the 118-bp segment, caused complete and partial loss, respectively, of methylation following paternal transmission in each transgenic mouse. Interestingly, these mutations overlap with the binding site for the transcription factor Kaiso, which is reportedly involved in maintaining paternal methylation at the human *H19* ICR (IC1) in cultured cells. In this study, we investigated if Kaiso regulates imprinted DNA methylation of the *H19* ICR in vivo.

**Results:**

Neither *Kaiso* deletion nor mutation of Kaiso binding sites in the 118-bp region affected DNA methylation of the mouse *H19* ICR transgene. The endogenous mouse *H19* ICR was methylated in a wild-type manner in *Kaiso*-null mutant mice. Additionally, the human IC1 transgene acquired imprinted DNA methylation after fertilization in the absence of Kaiso.

**Conclusions:**

Our results indicate that Kaiso is not essential for either post-fertilization imprinted DNA methylation of the transgenic *H19* ICR in mouse or for methylation imprinting of the endogenous mouse *H19* ICR.

**Supplementary Information:**

The online version contains supplementary material available at 10.1186/s13072-024-00544-8.

## Background

Genomic imprinting in mammals is an epigenetic phenomenon that causes parent-of-origin-specific monoallelic expression of specific genes. Most imprinted genes play crucial regulatory roles in development, growth, and behavior, and their dysregulation can lead to human disease [1, 2]. Imprinted genes occur in clusters and imprinting control regions (ICRs) have been identified as regions within these loci that exhibit allelic differences in DNA methylation, resulting in allele-specific patterns of gene expression. ICRs are germline differentially methylated regions (gDMRs) that undergo DNA methylation during either spermatogenesis or oogenesis. DNA methylation acquired from gametes is maintained in one allele after fertilization and throughout development [3, 4].

DNA methylation at ICRs undergoes cycles of erasure in primordial germ cells, establishment during sperm or oocyte formation, and maintenance after fertilization. Erasure and establishment are also observed in genomic sequences apart from ICRs. In contrast, maintenance occurs specifically at ICRs, particularly in preimplantation embryos immediately after fertilization, whereas methylation of most sequences in the genome is eliminated. During this period, methylation of the ICRs of the imprinted genes is maintained on only one allele. Additionally, as cell differentiation proceeds in the post-implantation embryo, one allele of the ICRs remains hypomethylated while various genomic sequences become DNA-methylated. A maintenance mechanism specific to ICRs has thus been proposed [5, 6].

At the *Igf2/H19* locus, the *H19* ICR, a gDMR that is DNA-methylated in sperm, has been identified in the intergenic region. After fertilization, this region is DNA methylated only on the paternal allele, resulting in the *Igf2* gene being expressed from the paternal allele while the *H19* gene is expressed from the maternal allele. Abnormalities in human *H19* ICR (hIC1) DNA methylation can cause Beckwith-Wiedemann syndrome and Silver-Russell syndrome [7–10].

We previously tested the activity of the mouse *H19* ICR in yeast artificial chromosome (YAC) transgenic mice (TgM), in which an *H19* ICR fragment (2.9 kb) was inserted into a YAC bearing the (non-imprinted) human β-globin locus [11]. We found that the transgenic *H19* ICR sequence was DNA-methylated in somatic cells of the offspring only when it was paternally inherited. However, it was not methylated in sperm and its paternal allele-specific methylation was acquired in a de novo methyltransferase-dependent manner at the one-cell stage embryo after fertilization [11, 12]. These results indicate that a post-fertilization allele-specific methylation mechanism (post-fertilization imprinted methylation) independent of sperm-derived methylation is responsible.

By generating transgenic mice with a series of deletion mutants, we determined that the 118-bp sequence within the *H19* ICR is necessary for post-fertilization methylation [12–15]. Deleting this sequence from the paternal endogenous *H19* ICR resulted in the loss of methylation in preimplantation embryos without altering its methylation status in sperm [15]. Therefore, the imprinted methylation activity after fertilization mediated by this 118-bp sequence is essential for maintaining the allelic methylation level of the mouse *H19* ICR.

This post-fertilization imprinted methylation was also observed in hIC1 transgenic mice [16]. In addition, the IG-DMR, which is the ICR of the other imprinted *Dlk1*-*Dio3* locus, also acquired paternal-origin-specific methylation immediately after fertilization in transgenic mice [17]. These results suggest that the post-fertilization imprinted methylation mechanism is conserved across species and imprinted genes.

The Krüppel-associated box (KRAB)-containing zinc finger proteins ZFP57 and ZFP445 are reportedly involved in maintaining DNA methylation in multiple ICRs, including the *H19* ICR [18–21]. These proteins bind to the ICRs in a CpG methylation-dependent manner. However, the mouse 118-bp sequence does not contain CpG [15]. In vitro binding experiments did not detect Zfp57 binding to the 118-bp sequence [14]. Therefore, other protein factors may regulate post-fertilization imprinted methylation via the 118-bp sequence.

Proteins that bind to the 118-bp sequence have been detected in nuclear extracts from P19 and ES cells [22]. In vitro binding experiments using mutant sequences showed that one of these proteins binds to five ‘RCTG’ motifs present in the 118-bp sequence. Transgenic mice were generated carrying the mouse *H19* ICR fragment with a total of five mutations, one within each RCTG motif. This mutant transgene remained unmethylated even when paternally transmitted. Moreover, deleting the 36-bp sequence from the mouse *H19* ICR transgene caused partial disruption of imprinted methylation; the 36-bp sequence is located at the 3ʹ end of the 118-bp sequence and lacks RCTG motifs [22]. Post-fertilization imprinted methylation in the mouse *H19* ICR is therefore likely to be induced by regulatory factors that bind to one or more motifs within the 118-bp sequence.

Kaiso (Zbtb33) is a zinc finger transcription factor of the pox virus and zinc finger (POZ) family that is associated with a range of cancers. Kaiso regulates epithelial-to-mesenchymal transition, apoptosis, and inflammation [23–25]. Kaiso binds to DNA by using its zinc fingers to recognize two consecutive methylated CpGs or a CpG-free motif (TNGCAG) called the Kaiso binding site (KBS) [26, 27]. Kaiso regulates transcription either negatively or positively by interacting with transcriptional regulatory protein complexes through its POZ domain [24,28–31]. Kaiso binding to the *H19* ICR in mouse brain was detected mainly on the paternally inherited allele [32]. Since Kaiso reportedly interacts with DNA methyltransferases Dnmt3a and Dnmt3b when overexpressed in cultured cells [33], it may induce DNA methylation at the imprinted loci. Importantly, Bohne et al. reported that when either KAISO was knocked down or the KBS in human IC1 was deleted from human primary fibroblasts, DNA methylation levels in paternally inherited IC1 were reduced, suggesting that KAISO is required to maintain hIC1 methylation [34].

We searched for a KBS sequence within the 118-bp sequence of the mouse *H19* ICR and found that one of the five nucleotides we identified as required for post-fertilization imprinted methylation is located within the KBS. Another KBS is found within the 36-bp sequence that was deleted from the aforementioned transgenic mouse. We therefore hypothesized that Kaiso induces imprinted methylation of the mouse *H19* ICR via KBSs in the 118-bp sequence. In this study, we tested this hypothesis by knocking out the *Kaiso* gene and introducing mutations in the KBS motifs within the 118-bp sequence of the mouse *H19* ICR transgene. Furthermore, we tested the requirement for Kaiso for acquisition of hIC1 methylation by determining the effect of Kaiso depletion on the hIC1 transgene methylation in vivo.

## Methods

### Mice

C57BL/6 J (B6) mice were purchased from Jackson Laboratory Japan. JF1/Msf (JF1) mice were provided by the RIKEN BRC through the National Bio-Resource Project of the MEXT and bred in the laboratory.

Transgenic mice carrying a human β-globin YAC with either the mouse *H19* ICR fragment or the human IC1 fragment inserted between the locus control region (LCR) and β-globin genes have been described previously [11, 16].

*Kaiso* gene knockout mice and ΔK-*H19* ICR YAC-TgM were generated using the *i*-GONAD method [35, 36]. To generate *Kaiso* knockout mice, two CRISPR RNAs (crRNAs) targeting the *Kaiso* gene sequence (5ʹ- ACCTGACTATTCGAAATGTG [AGG (PAM)] -3ʹ and 5ʹ -TGCAACTAGTCTACTTTCAG [AGG (PAM)] -3ʹ), trans-activating crRNA (tracrRNA) and Cas9 protein were introduced into fertilized eggs from wild-type C57BL/6 J mice. To generate ΔK-*H19* ICR YAC-TgM, crRNA targeting the Δ36-*H19* ICR YAC transgene sequence (5ʹ -CAGAACACACTTACATGGCA [TGG (PAM)] -3ʹ), tracrRNA, donor single-stranded oligodeoxynucleotides (5ʹ- GACCAAGGAAGCTTTCCTGCTCACTGTCCATTCAATGCAGTCAAAAGTGCTGTGACTATACAGGAGGAACATAGCA**t**TGCTGTGACCATACAGGAGGAACATAGCA**t**AGGCTAAAGGGCCATGGTGCCATGTAAGTGTGTTCTGTGCAGCAACTGATGACCAGACAGTACTGAGTCTGCCTGGAGCCTGAGTTAAAACCG -3ʹ, mutated nucleotides are bold, lower case letters) and Cas9 protein were introduced into fertilized eggs from Δ36-*H19* ICR YAC-TgM [22]. Tail DNA from founder progeny was screened by PCR amplification and sequencing. Individuals with the desired mutant alleles were crossed with wild-type animals to establish mutant lines.

### Preparation of oocytes and embryos

Female mice were super-ovulated via injection of CARD HyperOva (Kyudo), followed by human chorionic gonadotropin (hCG, Aska Pharmaceutical) (47–48-h interval). Unfertilized oocytes were collected from oviducts 18 h after hCG injection, and cumulus cells were removed by hyaluronidase treatment. Fertilized one-cell zygotes were collected from mated females 24 h after hCG injection, and cumulus cells were removed. Embryos at E3.5 (blastocysts) and E12.5 were obtained by natural mating.

### Electrophoretic mobility shift assay (EMSA)

**Fig. 1:**
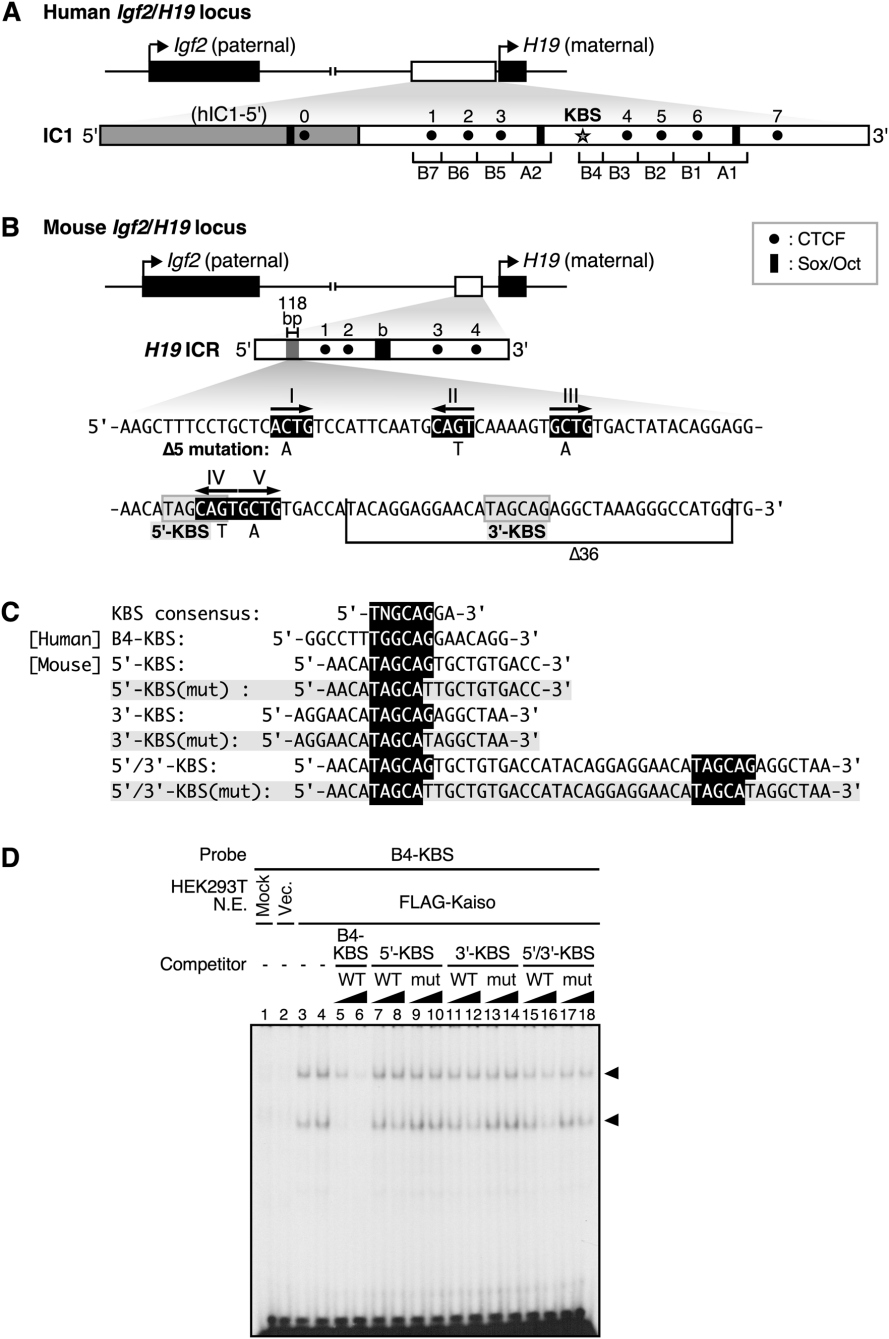
KBSs in human and mouse *H19* ICR. **A** Human *Igf2/H19* locus structure. The enlarged map shows the region of the human *H19* ICR (IC1) that we previously used as a transgene. Human IC1 has repetitive structures (A-repeats 1 and 2, and B-repeats 1–7) [39], with a KBS (indicated as a star) located within the B4 repeat. Dots (0–7) and black boxes indicate CTCF binding sites [40, 41] and Sox-Oct motifs [42, 43], respectively. **B** Mouse *Igf2/H19* locus structure. The enlarged map shows the region of the mouse *H19* ICR that we previously used as a transgene. Dots (1–4) indicate CTCF-binding sites [40, 41]. The black box indicates the 'b' region containing Sox-Oct motifs [43]. The nucleotide sequence of the 118-bp region is shown below the map. RCTG motifs I–V are shown in black and white inverted. Gray boxes indicate 5ʹ- and 3ʹ-KBSs. Mutated nucleotides in Δ5-*H19* ICR and the region deleted from Δ36-*H19* ICR mutants are indicated below the sequence [22]. **C** Nucleotide sequences of the KBSs in the human IC1 B4 repeat and in the mouse *H19* ICR 118-bp region. Double-stranded DNA fragments with these sequences were used either as a probe or as competitors in (**D**). **D** EMSA performed on nuclear extract from HEK293T cells overexpressing FLAG-tagged Kaiso protein and the B4-KBS probe. Three micrograms of nuclear extract were used in lane 3 and 7 μg in the others. Two hundred- and 800-times molar excess of competitors were used

Nuclear extracts were prepared from HEK293T cells overexpressing FLAG-tagged Kaiso protein using the Nuclear Extract Kit (Active Motif). Nuclear extracts (3 or 7 μg) were preincubated in the reaction mixture [PBS containing 5 mM MgCl_2_, 0.1 mM ZnSO_4_, 1 mM DTT, 0.1% NP40, 10% glycerol, and 1 μg poly(dI-dC)] with or without 200–800-times molar excess of a specific double-stranded competitor DNA for 10 min at RT. Radiolabeled DNA probe (15,000 cpm) was added and the incubation was continued for 25 min at RT. The incubation mixture was loaded on a 4% non-denaturing polyacrylamide gel in 0.5 × TBE buffer and electrophoresed at 4 ℃. The gels were subsequently dried and exposed to X-ray film. Probe and competitor sequences are shown in the Fig. 1C.

### Mouse ES cell establishment and culture conditions

The ES cell line was established from a blastocyst of an F1 hybrid formed between female JF1 and male B6 mice generated by in vitro fertilization (IVF). After culture in KSOM medium for about 4 days after IVF, an expanded blastocyst was placed on monolayers of mouse embryonic fibroblasts (MEFs), and cultured in DMEM (high glucose, pyruvate, Thermo Fisher) supplemented with 15% KnockOut Serum Replacement (Thermo Fisher), 1% fetal calf serum (FCS) (Roche), 1 × nonessential amino acids (Nacalai Tesque), 0.1 mM 2-mercaptoethanol (Nacalai Tesque), 50 U/ml penicillin–streptomycin (Thermo Fisher), 1000 U/ml mouse leukemia inhibitory factor (LIF) (Millipore), 0.2 µM PD0325901 (Wako), and 3 µM CHIR99021 (Wako) for 6 days. An inner cell mass outgrowth was transferred into 0.25% trypsin–EDTA (Thermo Fisher), dissociated by pipetting and plated on MEFs. At the time of this first passage, the medium was switched to ES medium, namely KnockOut DMEM (Thermo Fisher) supplemented with 15% FCS, 1 × nonessential amino acids, 2 mM L-glutamine (Nacalai Tesque), 0.1 mM 2-mercaptoethanol, 50 U/ml penicillin–streptomycin, and 1000 U/ml LIF. ES cell colonies were expanded to establish an ES cell line. Established ES cells were maintained on feeder MEFs with ES medium and used for further analysis.

### Chromatin immunoprecipitation (ChIP) assay

Chromatin immunoprecipitation (ChIP) was performed using a previously described procedure [37] with modifications. Approximately 3.3 million cells per IP were used. Trypsinized ES cells in ES medium were plated on gelatinized tissue culture dishes and incubated for 45 min at 37 ℃ and under 5% CO_2_ to remove MEFs. Medium containing unattached ES cells was recovered and cells were collected by centrifugation. Cells were resuspended in PBS, fixed with 1% formaldehyde for 10 min at room temperature, and quenched with 250 mM Tris–HCl pH 8.0 for 10 min at room temperature. Cells were washed once with cold PBS supplemented with 1 mM PMSF and cell pellets were snap-frozen in liquid nitrogen before being stored at − 80 ℃. Cell pellets were quickly defrosted and resuspended in nuclear extraction buffer (50 mM Tris–HCl pH 7.4, 140 mM NaCl, 1 mM EDTA, 10% glycerol, 0.5% Nonidet P-40, protease inhibitor cocktail (Nacalai Tesque)) for 10 min on ice. After centrifugation, supernatants were discarded and pellets were resuspended in ChIP lysis buffer (50 mM Tris–HCl pH 8.0, 140 mM NaCl, 1 mM EDTA, 10% glycerol, 1% Triton X-100, 0.5% SDS, protease inhibitor cocktail). Samples were incubated for 20 min on ice and sonicated with a Picoruptor sonication device (Diagenode). After sonication, cell debris was removed by centrifugation and supernatants were used for subsequent experiments. After verifying that chromatin was fragmented into 100–800 bp pieces, samples containing 25 µg DNA were collected and diluted 1:5 in IP dilution buffer (10 mM Tris–HCl pH 8.0, 140 mM NaCl, 1 mM EDTA, 1% Triton X-100, protease inhibitor cocktail). The samples were pre-incubated with Dynabeads protein G (Thermo Fisher) for 1 h at 4 ℃ with rotation, and the beads later removed. Next, either normal rabbit IgG (sc-2027, Santa Cruz), Kaiso/ZBTB33 rabbit polyclonal antibody (A12900, ABclonal), or CTCF rabbit monoclonal antibody (3418S, Cell Signaling) was added to the sample at 1:100 dilution and incubated overnight at 4 ℃ with rotation. After antibody incubation, Dynabeads protein G was added to the samples, and they were incubated for 6 h at 4 ℃ with rotation. The beads were washed four times with wash buffer (10 mM Tris–HCl pH 8.0, 140 mM NaCl, 1 mM EDTA, 1% glycerol, 0.5% Triton X-100, 0.01% SDS) and twice with LiCl buffer (10 mM Tris–HCl pH 8.0, 1 mM EDTA, 250 mM LiCl, 0.5% Triton X-100). After proteinase K treatment and decrosslinking in elution buffer (10 mM Tris–HCl pH 8.0, 300 mM NaCl, 5 mM EDTA, 1% SDS, 0.8 mg/ml proteinase K) for 1 h at 55 ℃ and overnight at 65 ℃, respectively, DNA was purified using a QIAquick PCR purification Kit (Qiagen). Quantitative amplification of DNA was performed with a Thermal Cycler Dice (TaKaRa Bio) using TB Green Premix EX Taq II (TaKaRa Bio). The allelic ratio in qPCR products was determined by pyrosequencing using SNPs between JF1 (maternal allele) and B6 (paternal allele) and a Pyromark Q24 system (Qiagen). Primers used for qPCR and pyrosequencing are listed in Table 1.

**Table 1:** Primers for ChIP-qPCR and pyrosequencing

Primer sets for qPCR and pyrosequencing			
Region analyzed	5ʹ primer	3ʹ primer	Sequencing primer
Region-I	(5ʹ biotin) H19ICR-SNP-F5	H19ICR-SNP-R5	H19ICR-SNP-S5new
Region-II	H19ICR-SNP-F3	(5' biotin) H19ICR-SNP-R3	H19ICR-SNP-S3

Primer sequences	
Primer	Sequence
H19ICR-SNP-F5	5ʹ- TGCTGCACAGAACACACTTACAT -3ʹ
H19ICR-SNP-R5	5ʹ- CCCATCGAAATGCAAATGAAC -3ʹ
H19ICR-SNP-S5new	5ʹ- TTCCTGCTCACTGTCC -3ʹ
H19ICR-SNP-F3	5ʹ- CACGCGGCAGTTTCTATGT -3ʹ
H19ICR-SNP-R3	5ʹ- CTTCTGCCTTGGGACTCCTC -3ʹ
H19ICR-SNP-S3	5ʹ- CTCCCGCCTATAACC -3ʹ

### DNA methylation analysis

Genomic DNA extracted from either tail tips of ~ 1-week-old animals or adult sperm was digested with *Xba*I and treated with sodium bisulfite using the EZ DNA Methylation Kit (Zymo Research). Blastocysts were embedded in agarose beads and treated with sodium bisulfite as described previously [12]. Subregions of transgenic mouse *H19* ICR, endogenous mouse *H19* ICR, and transgenic human IC1 were amplified by nested or single-round PCR. PCR products were subcloned into the pGEM-T Easy vector (Promega) for Sanger sequencing. Sequencing results were analyzed with Quantification tool for Methylation Analysis (QUMA, http://quma.cdb.riken.jp). Alternatively, subregions of transgenic and endogenous mouse *H19* ICR, which were amplified by nested or single-round PCR, were subjected to pyrosequencing using a Pyromark Q24 system. PCR primers are listed in Table 2.

**Table 2:** Primers for DNA methylation analysis

Primer sets for bisulfite Sanger sequencing				
Region analyzed	PCR round		5ʹ primer	3ʹ primer
Mouse *H19* ICR (wild-type and ΔK) transgene	Nested PCR	1st	ICR-MA-5S13	BGLB-MA-3A2
		2nd	ICR-MA-5S13	ICR-MA-3A2
Mouse endogenous *H19* ICR	Single-round PCR		ICR-MA-5S13	ICR-MA-3A2
Human IC1 transgene	Single-round PCR		hIC1-MA-5S4	hIC1-MA-3A1

Primer sets for bisulfite pyrosequencing					
Region analyzed	PCR round		5ʹ primer	3ʹ primer	Sequencing primer
Mouse *H19* ICR (wild-type) transgene	Nested PCR	1st	ICR-MA-5S13	BGLB-MA-3A2	
		2nd	ICR-MA-5S26	(5' biotin) ICR-MA-3A31	ICR-MA-5S26
Mouse endogenous *H19* ICR (blastocyst)	Nested PCR	1st	ICR-MA-5S13	ICR-MA-3A2	
		2nd	ICR-MA-5S26	(5' biotin) ICR-MA-3A31	ICR-MA-5S26
Mouse endogenous *H19* ICR (tail)	Single-round PCR		ICR-MA-5S26	(5' biotin) ICR-MA-3A31	ICR-MA-5S26
Mouse endogenous IG-DMR	Single-round PCR		mIGDMR-MA-5S3	(5' biotin) mIGDMR-MA-3A2	mIGDMR-MA-5S4

Primer sequences		
Region analyzed	Primer	Sequence
Mouse *H19* ICR	ICR-MA-5S13	5ʹ- GGTGATTTATAGTATTGTTATTTG -3ʹ
	BGLB-MA-3A2	5ʹ- TTCTAACCCCACAAAAATTTATTC -3ʹ
	ICR-MA-3A2	5ʹ- AACAATACTAAATCTACCTAAAAC -3ʹ
	ICR-MA-5S26	5ʹ- GGGGGGGTTTTTTAGGTTTGG -3ʹ
	ICR-MA-3A31	5ʹ- CTACAAAAAAACCATACCCTATTCTT -3ʹ
Human IC1	hIC1-MA-5S4	5ʹ- TATTTGGGTTTTGTTAGTTTTTTG -3ʹ
	hIC1-MA-3A1	5ʹ- CTCCTTCCATCTCACTACTCTAAA -3ʹ
Mouse IG-DMR	mIGDMR-MA-5S3	5ʹ- TTAGGAGTTAAGGAAAAGAAAGAAATAG -3ʹ
	mIGDMR-MA-3A2	5ʹ- ATTTATCATAAACAAATCCCATAACTTACT -3ʹ
	mIGDMR-MA-5S4	5ʹ- GTTAAGGAAAAGAAAGAAATAGT -3ʹ

### RT-PCR

Total RNA of MII oocytes, one-cell zygotes, and livers at E12.5 was extracted by ISOGEN (Nippon Gene) and converted to cDNA using ReverTra Ace qPCR RT Master Mix with gDNA Remover (TOYOBO). Quantitative amplification of cDNA was performed with the Thermal Cycler Dice using TB Green Premix EX TaqII and PCR primers listed in Table 3.

**Table 3:** Primers for expression analysis

Gene	Primer	Sequence
*Kaiso*	mKAISO-seq-5S1	5ʹ- TAGGACTCTGACCCTGCCTCG -3ʹ
	mKAISO-seq-3A1	5ʹ- ACGCTGTTCATTCAAGGAGTTCA -3ʹ
*H19*	mH19-5S2	5ʹ- CGGTGTGATGGAGAGGACAGAAG -3
	mH19-3A2	5ʹ- CCAGAGAGCAGCAGGCAAGTGTTAG -3ʹ
*Igf2*	mIgf2-5S2	5ʹ- TCTGTGCGGAGGGGAGCTTGTT -3ʹ
	mIgf2-3A2	5ʹ- CAGCACTCTTCCGCGATGCCAC -3ʹ
*Gapdh*	mGAPDH-5S	5ʹ- AAAATGGTGAAGGTCGGTGTG -3ʹ
	mGAPDH-3A	5ʹ- TGAGGTCAATGAAGGGGTCGT -3ʹ

## Results

### Kaiso as a candidate regulator of mouse *H19* ICR DNA methylation

The B4 repeat of the human *H19* ICR (hIC1) contains a KBS (Fig. 1A) that is necessary for maintaining DNA methylation of paternally inherited hIC1 in human primary fibroblasts [34]. We previously found that post-fertilization imprinted methylation of mouse *H19* ICR is mediated by a 118-bp sequence [15]. A five-nucleotide mutation in the RCTG motifs (I–V, Fig. 1B) within the 118-bp sequence disrupts in vitro binding to the protein in nuclear extracts, as well as post-fertilization imprinted methylation in transgenic mice (Δ5-*H19* ICR) [22]. We found one KBS, designated 5ʹ-KBS, which overlaps with the RCTG motif IV, and another, designated 3'-KBS, in the downstream region. Deletion of this region also caused partial disruption of imprinted methylation in transgenic mice (Δ36-*H19* ICR, Fig. 1B) [22]. Therefore, we hypothesized that the loss of DNA methylation in paternally inherited Δ5- and Δ36-*H19* ICR transgenes is due to the inhibition of Kaiso action mediated by the KBSs.

First, we determined whether Kaiso can bind to KBSs in the mouse 118-bp sequence by EMSA (Fig. 1C, D). Our results indicate that Kaiso protein overexpressed in HEK293T cells bound to a ^32^P-labeled probe containing the KBS from the hIC1 B4-repeat (B4-KBS). Adding an unlabeled B4-KBS fragment as a competitor resulted in the loss of Kaiso binding to the probe, confirming the specificity of the reaction. Next, we used fragments containing KBSs from the mouse 118-bp sequence as competitors in the binding assay. Our results indicate that either 5ʹ- or 3ʹ-KBS alone had no effect. However, when a fragment containing both KBSs was used, Kaiso binding to B4-KBS was partially inhibited. Fragments with mutations introduced into the KBSs had no competitive effect. Thus, Kaiso appears to bind weakly to the KBSs from the 118-bp sequence of the mouse *H19* ICR. Moreover, the results of these binding experiments, in which Kaiso bound when both 5ʹ- and 3ʹ-KBSs were present, suggest that Kaiso cannot maintain DNA methylation, if any, of the mouse *H19* ICR sequence when one of the KBS sites is mutated. Our previous results showing complete loss of methylation in the Δ5-*H19* ICR transgene with disrupted 5ʹ-KBS and partially reduced methylation in the Δ36-*H19* ICR transgene with deleted 3ʹ-KBS [22] are consistent with our hypothesis that Kaiso binds to the *H19* ICR via KBSs within the 118-bp sequence and regulates DNA methylation.

Post-fertilization imprinted methylation of the mouse *H19* ICR is initiated in the early embryos immediately after fertilization and may involve proteins from oocytes. However, due to the limited sources available, it is very challenging to verify the binding of specific proteins to genomic DNA at such time points. Therefore, we sought to determine if Kaiso can bind to the mouse *H19* ICR sequence in ES cells using chromatin immunoprecipitation (ChIP). We used an ES cell line established from a blastocyst obtained through in vitro fertilization of mice of different strains (JF1/Msf oocytes and C57BL/6 J sperm) so that the parental origin of each allele could be distinguished by SNPs (Fig. S1A).

Kaiso has been reported to bind to methylated CGCGs or CG-free KBSs [26, 27]. Consistent with the presence of multiple CGCGs and KBSs within the mouse *H19* ICR sequence (Fig. S1A), Kaiso enrichment was detected at both the 118 bp and downstream portion of the *H19* ICR (Fig. S1B). Kaiso thus appears to bind to both alleles, although slightly more frequently to the paternal allele (Fig. S1B), which may be due to its interaction with methylated CGCG motifs on the paternal allele and KBSs on both alleles.

### Effect of *Kaiso* deletion on mouse *H19* ICR DNA methylation in vivo

**Fig. 2:**
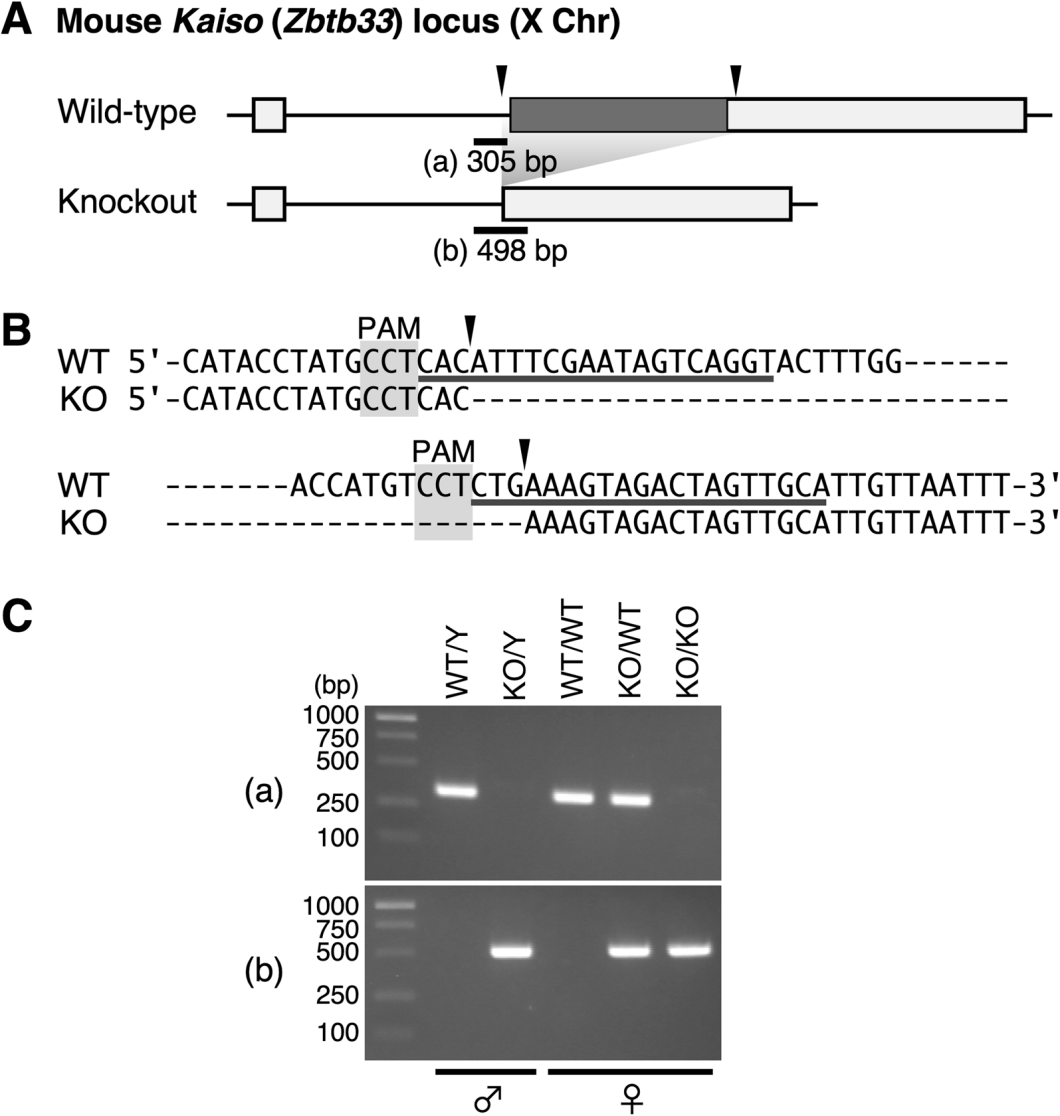
Generation of *Kaiso* gene knockout mice. **A** Structure of the mouse *Kaiso* gene locus on chromosome X. Cas9 target sites for generating the *Kaiso* gene knockout allele are indicated with arrowheads. Targeting two sites removes the *Kaiso* gene’s coding sequence. Positions and sizes of PCR amplicons used to distinguish wild-type (a﻿) from knockout (b) alleles in (c) are shown. **B** Sequence alignment of wild-type (WT) and knockout (KO) *Kaiso* alleles. Protospacer-adjacent motif (PAM) and gRNA sequences are shaded and underlined, respectively. Cleavage sites predicted from PAM locations are indicated with arrowheads. **C** Tail DNA from mice of each genotype was subjected to PCR analysis using the primer sets shown to the left of each panel

We proceeded to determine if Kaiso regulates DNA methylation of the mouse *H19* ICR in vivo. To this end, crRNAs and a tracrRNA targeting both sides of the coding sequence of the *Kaiso* gene, which is located on the X chromosome (Fig. 2A, B), in addition to Cas9 protein, were introduced into fertilized mouse eggs. The resulting mice lack the *Kaiso* coding sequence (Fig. 2C). We also verified that the *Kaiso* gene transcript variants 1 and 2, which were detected in wild-type MII oocytes and 1-cell zygotes, were lost in *Kaiso* knockout embryos (Fig. S2). As previously reported [23], these *Kaiso*-null mice were viable and fertile with no detectable abnormalities.

**Fig. 3:**
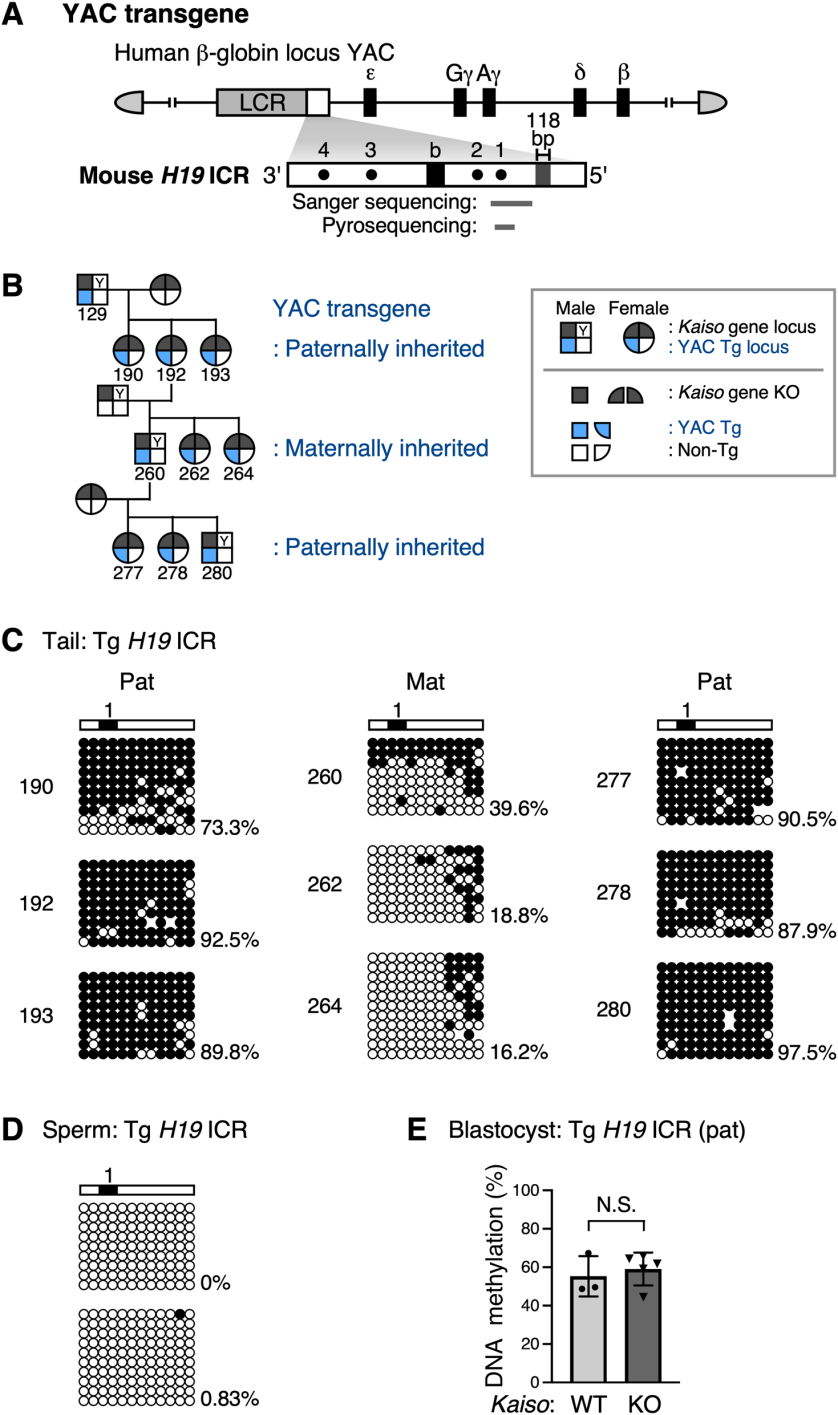
DNA methylation status of transgenic mouse *H19* ICR in *Kaiso* knockout mice. **A** *H19* ICR/β-globin YAC transgene structure. The 150-kb human β-globin locus YAC carries the LCR (gray box) and β-like globin genes (black boxes). The 2.9-kb mouse *H19* ICR fragment (inverted orientation) was introduced between the LCR and β-globin genes. Gray bars below the map indicate the sequences determined by bisulfite Sanger sequencing in (**C**) and (**D**), and by bisulfite pyrosequencing in (**E**). **B** Pedigree of individuals whose transgenic *H19* ICR DNA methylation status was determined in (**C**). **C** DNA methylation status of transgenic *H19* ICR under Kaiso deficiency conditions. The DNA methylation status of the paternally (Pat) or maternally (Mat) inherited transgenic *H19* ICR in tail somatic cells of individuals indicated in (**B**) was determined by bisulfite sequencing. Each horizontal row represents a single DNA template molecule. Methylated and unmethylated CpG motifs are shown as filled and open circles, respectively. Position of CTCF-binding sites is shown by filled boxes. **D** DNA methylation status of transgenic *H19* ICR in *Kaiso* deficiency sperm was determined by bisulfite sequencing. **E** DNA methylation status of paternally inherited transgenic mouse *H19* ICR in blastocysts in the presence (WT) or absence (KO) of *Kaiso*. Blastocyst stage embryos from a single litter were pooled and used as the sample; three *Kaiso* WT litters and five *Kaiso* KO litters were analyzed by bisulfite pyrosequencing. The mean methylation level and standard deviation are shown. The statistical significance of observed differences was determined using an unpaired *t-*test (*N.S*. not statistically significant)

To analyze the methylation state of the transgenic *H19* ICR under Kaiso-deficient conditions, *Kaiso* knockout mice were crossed with *H19* ICR YAC-TgM to generate individuals that both carry the transgene and are deficient in Kaiso. In somatic cells, the paternally derived transgenic *H19* ICR was highly methylated to the same extent as in the presence of Kaiso [11, 22], whereas the maternally inherited transgenic *H19* ICR was hypomethylated (Fig. 3A–C). Furthermore, the maternally inherited hypomethylated transgenic *H19* ICR became highly methylated when passed through males, indicating reprogramming of imprinting (Fig. 3A–C).

In the presence of Kaiso, the transgenic *H19* ICR remains unmethylated in sperm and acquires paternal allele-specific methylation after fertilization [12]. In the absence of Kaiso, the transgenic *H19* ICR was also found to be unmethylated in sperm (Fig. 3D). At the blastocyst stage, the paternally inherited transgenic *H19* ICR was methylated to the same extent in *Kaiso*-null embryos as in wild-type embryos (Fig. 3E). Therefore, the methylation of the transgenic *H19* ICR likely occurs after fertilization in a Kaiso-independent manner.

**Fig. 4:**
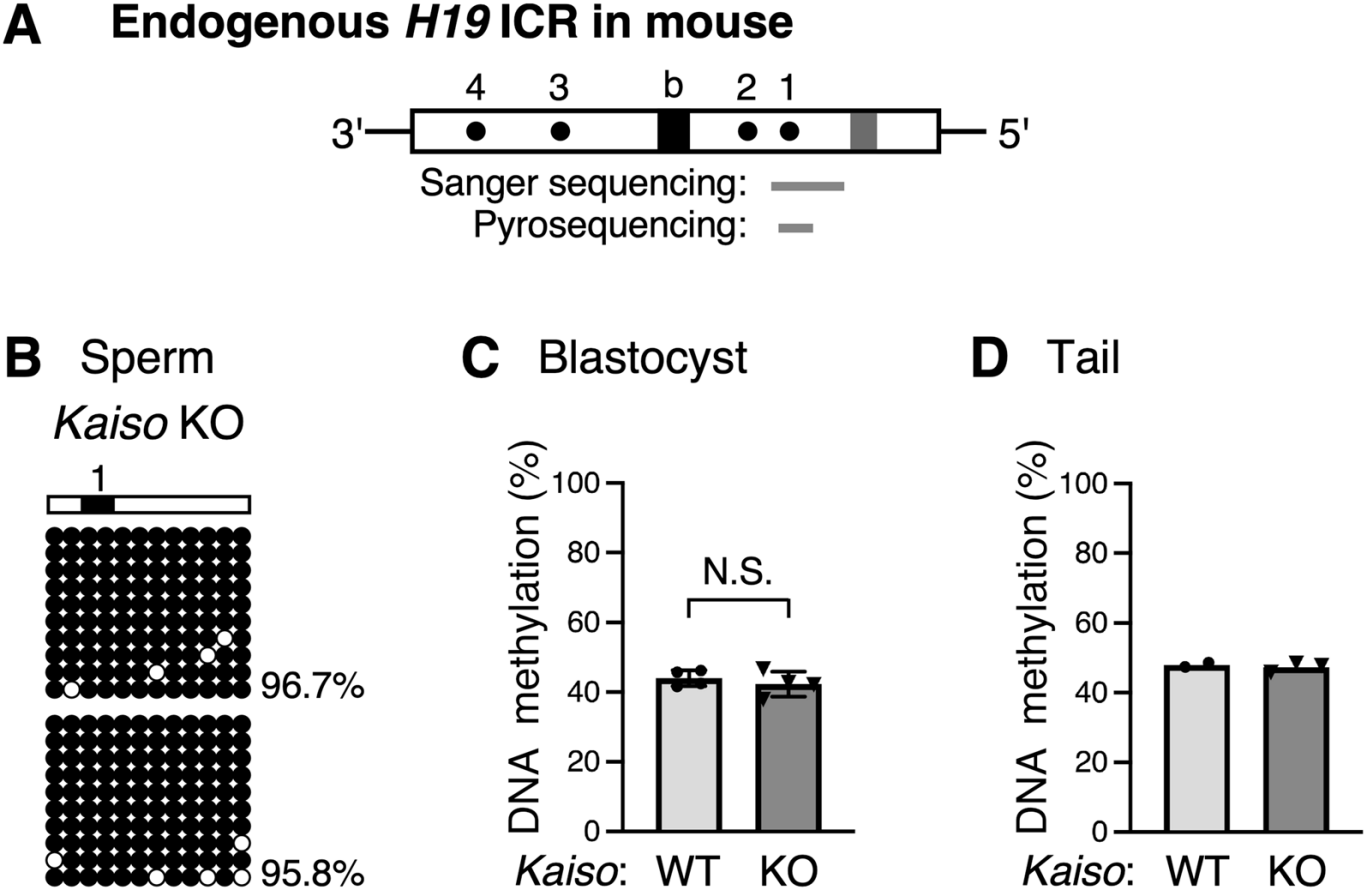
DNA methylation status of endogenous *H19* ICR in *Kaiso* knockout mice. **A** Map of the endogenous mouse *H19* ICR fragment. Regions analyzed by bisulfite Sanger sequencing or pyrosequencing in (**B**–**D**) are shown as gray bars below the map. **B** DNA methylation status of *H19* ICR in *Kaiso* deficiency sperm was determined by bisulfite Sanger sequencing. **C** DNA methylation status of *H19* ICR in blastocysts in the presence (WT) or absence (KO) of *Kaiso*. Blastocyst stage embryos from a single litter were pooled and used as the sample; each four *Kaiso* WT or KO litters were analyzed by bisulfite pyrosequencing. The average and standard deviation are depicted. Statistical differences were determined using an unpaired *t-*test (*N.S.* not significant). **D** DNA methylation status of endogenous mouse *H19* ICR in somatic cells in the presence (WT) or absence (KO) of *Kaiso* was analyzed by bisulfite pyrosequencing. Tail somatic cell DNA from two *Kaiso* WT individuals and three *Kaiso* KO individuals was analyzed

Next, we determined the methylation status of the endogenous mouse *H19* ICR. The endogenous *H19* ICR was highly methylated in sperm from Kaiso-deficient mice, just as in wild-type mice (Fig. 4A, B). By analyzing both parental alleles in blastocyst stage embryos and tail somatic cells of wild-type mice, we found DNA methylation level of the endogenous *H19* ICR was approximately 50%, consistent with hypermethylation on the paternal allele and hypomethylation on the maternal allele. Kaiso deletion also resulted in approximately 50% methylation in both cell types (Fig. 4C, D, Fig. S3A-C). Therefore, Kaiso does not appear to affect the differential methylation of the endogenous *H19* ICR. Consistent with these observations, the loss of Kaiso did not cause a clear change in the transcript levels of the *Igf2* and *H19* genes (Fig. S4).

Taken together, these results suggest that Kaiso is not required for paternal allele-specific methylation of the mouse *H19* ICR.

### Effect of KBS mutation on DNA methylation of the mouse *H19* ICR transgene

**Fig. 5:**
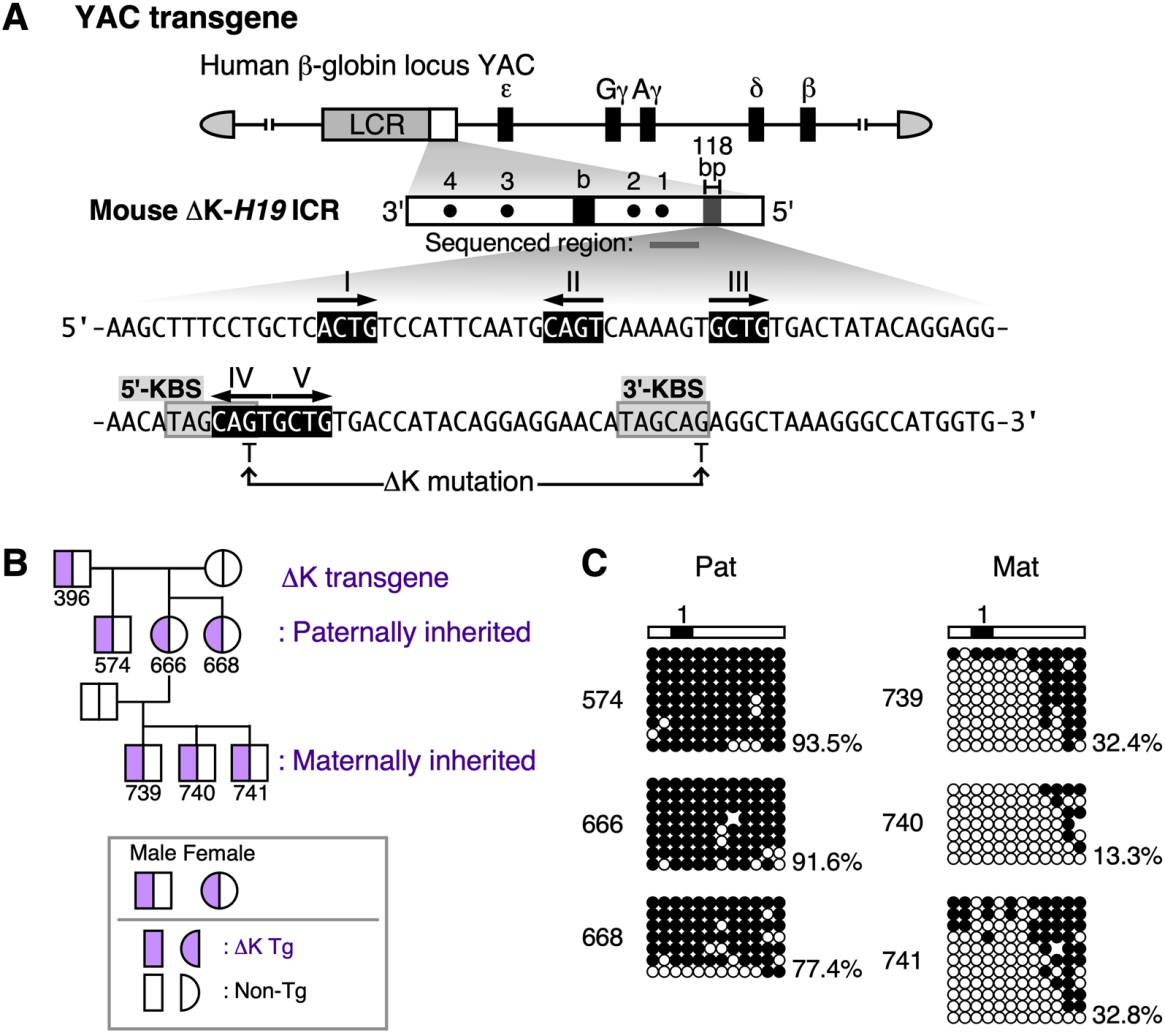
DNA methylation status of transgenic mouse *H19* ICR fragment with KBS mutations. **A** Generation of YAC-TgM with KBS mutations in transgenic mouse *H19* ICR fragment. Structure of the YAC transgene and mutated nucleotides within the 118-bp sequence in the transgenic mouse *H19* ICR fragment are shown. Gray boxes indicate 5ʹ- and 3ʹ-KBSs. RCTG motifs I-V are shown in black and white inverted. **B** Pedigree of individual mice whose ΔK-*H19* ICR DNA methylation status was determined in (**C**). **C** DNA methylation status of the paternally (Pat) or maternally (Mat) inherited ΔK-*H19* ICR transgene in the tail somatic cells of the individuals in (**B**) was determined by bisulfite sequencing

In addition to Kaiso, another POZ family zinc finger protein, Zbtb4, has been found to bind to KBS [32]. Therefore, the acquisition of paternal-specific methylation by the mouse *H19* ICR in the absence of Kaiso may be due to compensation by Zbtb4. To investigate this possibility, we introduced mutations at the 5'- and 3'-KBSs of the mouse *H19* ICR (Fig. 5A, ΔK-*H19* ICR). Analysis of the methylation status in YAC-TgM revealed that the paternally inherited ΔK-*H19* ICR was hypermethylated, whereas the maternally inherited one was hypomethylated (Fig. 5B, C), as observed for the wild-type transgene. Thus, it is likely that Kaiso and Zbtb4 are not required for regulating DNA methylation in the mouse *H19* ICR. In addition, the imprinted methylation of the ΔK-*H19* ICR, in contrast to the complete loss of methylation of the Δ5-*H19* ICR observed in our previous study [22], suggests that RCTG motifs apart from RCTG-IV are sufficient for methylation acquisition by the mouse *H19* ICR.

### DNA methylation status of human IC1 transgene in Kaiso-deficient embryos

The results described above indicate that Kaiso is not required for imprinted methylation of the mouse *H19* ICR sequence. However, Bohne et al*.* have reported that Kaiso is required for IC1 methylation in human primary fibroblasts [34]. Our previous experiments have indicated that hIC1 also exhibits post-fertilization imprinted methylation activity when inserted into the human β-globin YAC transgene [16]. We thus investigated the potential role of Kaiso in regulating imprinted methylation in hIC1 YAC-TgM.

**Fig. 6:**
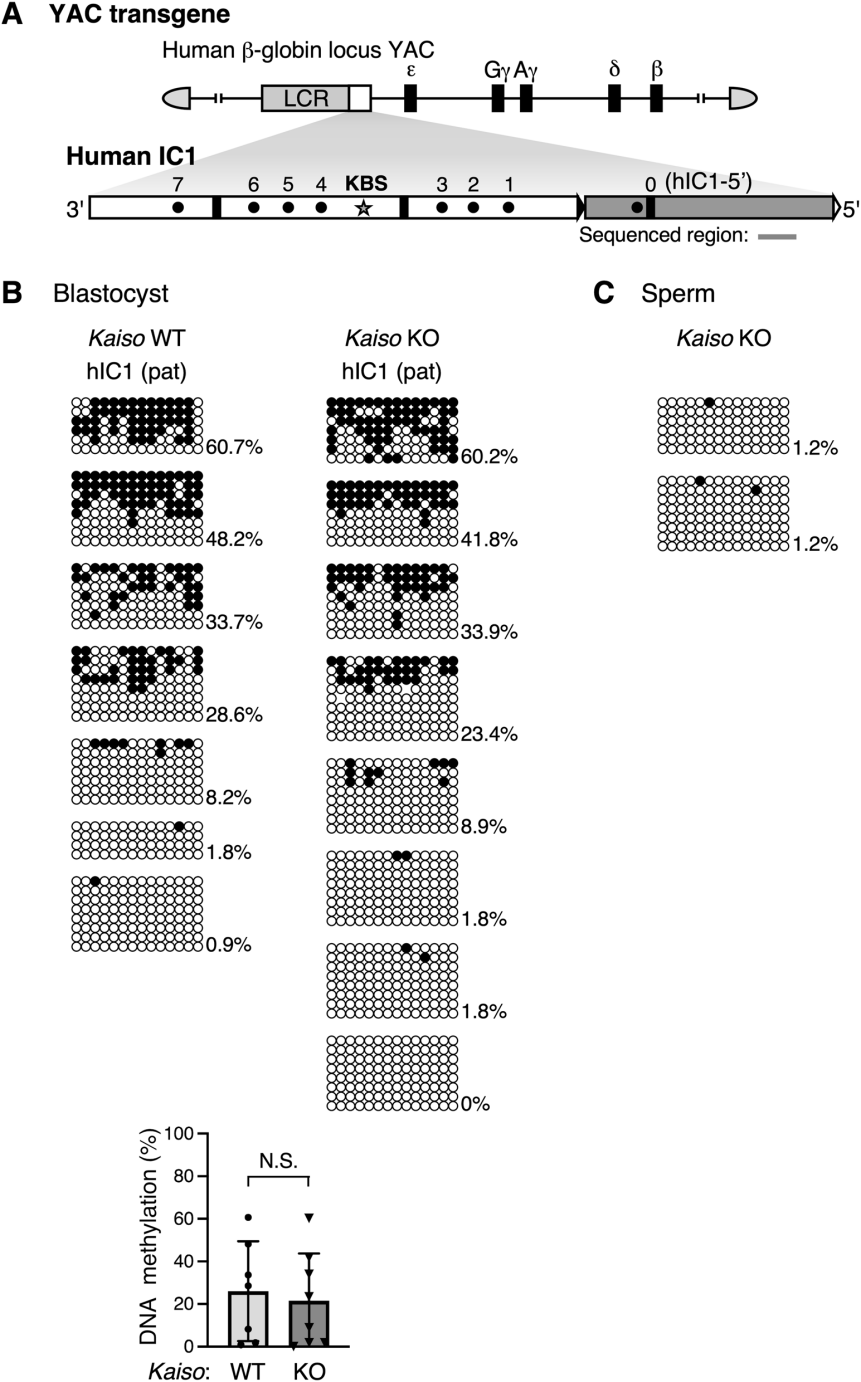
DNA methylation status of transgenic human IC1 in *Kaiso* knockout mice. **A** Structure of YAC transgene in which human IC1 fragment was introduced between the LCR and β-globin genes. Gray bar below the map indicates the sequence analyzed by bisulfite sequencing in (**B**) and (**C**). **B** DNA methylation status of paternally inherited transgenic human IC1 in blastocysts in the presence (WT) or absence (KO) of *Kaiso*. Blastocyst stage embryos from one litter were used as the sample; seven *Kaiso* WT litters and eight *Kaiso* KO litters were analyzed by bisulfite sequencing. The average and standard deviation of DNA methylation level of each genotype are displayed on the graph. Statistical significance of observed differences was determined using an unpaired *t-*test (*N.S.* not significant). **C** DNA methylation status of transgenic human IC1 in Kaiso deficiency sperm was determined by bisulfite sequencing

Post-fertilization methylation of hIC1 in TgM is detected in preimplantation embryos in the upstream region of hIC1 after paternal, but not maternal, transmission [16]. Therefore, we determined the methylation status of the hIC1 transgene in *Kaiso*-deficient blastocysts (Fig. 6A, B). Our results showed variations in DNA methylation levels among different embryos (litters), but no significant methylation changes were observed when compared to wild-type embryos that express Kaiso. In addition, since the hIC1 transgene was not methylated in sperm when Kaiso was deleted (Fig. 6C), the methylation detected in blastocysts must be acquired after fertilization. Therefore, Kaiso is not required for the acquisition of post-fertilization imprinted methylation at the hIC1 transgene.

## Discussion

Our previous studies have shown that post-fertilization imprinted methylation of the mouse *H19* ICR requires all or some of the five nucleotides we deleted from the 118-bp sequence [22]. This strongly suggests that DNA methylation of the *H19* ICR may be regulated by protein factors that recognize and bind to specific sequences containing these five nucleotides. In this study, we focused on Kaiso as a candidate and tested its requirement in the regulation of DNA methylation at the *H19* ICR by knocking out the *Kaiso* gene. We found that Kaiso is not required for the acquisition of post-fertilization paternal allele-specific DNA methylation of the mouse transgenic *H19* ICR sequence. We also found that Kaiso is not required for germline establishment and post-fertilization maintenance of methylation imprints at the endogenous mouse *H19* ICR. Although Zbtb4, like Kaiso, can also bind to KBSs [32], Zbtb4 is unlikely to compensate for *Kaiso* gene function because Zbtb4 expression was not detected in oocytes [38], which provide the DNA methylation machinery found in one-cell zygotes, within which post-fertilization imprinted methylation of the *H19* ICR occurs. In accordance, DNA methylation levels were not altered by mutations in the KBSs (ΔK) within the transgenic *H19* ICR. Therefore, other proteins may play a role as factors that bind to the mouse *H19* ICR sequence and induce methylation.

Bohne et al*.* reported that KAISO is required for the maintenance of human IC1 DNA methylation in human primary fibroblasts [34]. In this study, we tested the requirement for Kaiso in human IC1 transgenic mice. However, *Kaiso* deficiency did not affect the level of post-fertilization imprinted methylation of human IC1 in preimplantation embryos. We therefore concluded that Kaiso is not required for methylation acquisition during this period. On the other hand, human IC1 transgene methylation in the preimplantation embryo is only detected in regions away from the KBS, and methylation near the KBS is not recapitulated in transgenic mice [16]. Therefore, our results do not rule out the possibility that KAISO is required to maintain methylation near the KBS in human cells. This will require future verification.

The KBS in the B4 repeat of human IC1 contains the optimal, eight-nucleotide binding motif (TNGCAGGA) [27, 34]. In contrast, the two KBSs within the 118-bp sequence of the mouse *H19* ICR contain only the core sequences (TNGCAG). In the entire 2.9 kb *H19* ICR, there are also multiple KBS core sequences, but no complete eight-nucleotide motifs. This difference may influence the difference in dependence on Kaiso for maintaining DNA methylation between human and mouse *H19* ICR. In the mouse IG-DMR fragment, in which post-fertilization imprinted methylation was observed in our previous transgenic mouse experiments, as in the mouse *H19* ICR [17], several KBS core sequences are present, but the complete eight-nucleotide motif is absent. Therefore, Kaiso is not expected to be required for differential methylation of the mouse IG-DMR. Indeed, analysis of endogenous IG-DMR in Kaiso-deficient mice showed no changes in methylation levels (Fig. S5).

The ΔK-*H19* ICR sequence correctly acquired imprinted DNA methylation, indicating that of the five nucleotides we previously identified, C in RCTG motif-IV is not required for methylation. Since post-fertilization imprinted methylation of the *H19* ICR does not require DNA methylation in the sperm, a still undetermined sperm-derived epigenetic mark deposited on the *H19* ICR is likely used to identify the parental allele after fertilization. Therefore, there may be a factor that recognizes the above four nucleotides to add this mark during spermatogenesis. Alternatively, since imprinted methylation of the *H19* ICR in the one-cell stage embryo after fertilization is initiated by the oocyte-derived de novo DNA methyltransferases Dnmt3a and Dnmt3L [12], oocyte-originating factors that bind to the above four nucleotides may help recruit de novo methyltransferases. Identifying binding factors for the four nucleotides in the *H19* ICR in the male germ line or oocyte will therefore be crucial for uncovering how post-fertilization imprinted methylation of the *H19* ICR is regulated.

## Conclusions

We have demonstrated that *Kaiso* deletion did not affect the DNA methylation status of either transgenic or endogenous mouse *H19* ICR, or transgenic human IC1 sequences. The mouse *H19* ICR transgene carrying mutations of KBSs in the 118-bp sequence becomes methylated after fertilization, similarly to the wild-type transgene. These findings suggest that Kaiso is not necessary for either post-fertilization imprinted methylation of the transgenic *H19* ICR or for methylation imprinting of the endogenous mouse *H19* ICR.

### Supplementary Information

Supplementary Material 1: Figure S1 Kaiso recruitment to endogenous mouse *H19* ICR. **A** Mouse *H19* ICR structure. Dots (1–4) indicate CTCF-binding sites. The black box indicates the 'b' region containing Sox-Oct motifs. Gray bars below the map indicate the regions amplified by qPCR (left, in each panel in B), which include SNPs analyzed by pyrosequencing (right, in each panel in B). **B** (Left in each panel) Chromatin from JF1/B6 hybrid mouse ES cells was immunoprecipitated using either control IgG, anti-Kaiso, or CTCF antibodies. Following qPCR analyses of the regions indicated in (A), relative enrichment values (Kaiso/IgG or CTCF/IgG signal ratios) were calculated. (Right in each panel) qPCR products were analyzed by pyrosequencing to determine binding allelic ratio using SNPs between JF1 (maternal allele) and B6 (paternal allele). Input chromatin was analyzed simultaneously as a control. CTCF bound more preferentially to the maternal allele than to the paternal allele, as previously reported [40, 41, 44].

Supplementary Material 2: Figure S2 *Kaiso* expression in oocytes and embryos. **A** Transcripts from *Kaiso* gene locus. Coding sequences in exons (rectangles) are indicated with filled boxes. Cas9 target sites for generating *Kaiso* gene knockout allele are indicated with arrowheads. cDNA regions amplified by PCR in (B) are indicated below the maps. **B** Total RNA was extracted from WT or *Kaiso* KO oocytes (30, 32, or 26 oocytes) and one-cell zygotes (31 or 12 embryos, respectively), reverse-transcribed, and the resulting cDNA was subjected to PCR using primer sets for either *Kaiso* or *Gapdh*.

Supplementary Material 3: Figure S3 DNA methylation status of endogenous *H19* ICR in *Kaiso* knockout mice. **A** Map of the endogenous mouse *H19* ICR fragment. The region analyzed by bisulfite Sanger sequencing in (B) and (C) is shown as a gray bar below the map. **B** DNA methylation status of *H19* ICR in blastocysts in the presence (WT) or absence (KO) of *Kaiso*. Blastocyst stage embryos from a single litter were pooled and used as the sample. Each of four *Kaiso* WT or KO litters that were also analyzed by pyrosequencing in Fig. 4C were analyzed by Sanger sequencing. Bars under columns indicate CpGs analyzed by pyrosequencing in Fig. 4C. **C** DNA methylation status of *H19* ICR in tail somatic cells from *Kaiso* WT or KO individuals that were also analyzed in Fig. 4D was determined by bisulfite Sanger sequencing. Bars under columns indicate CpGs analyzed by pyrosequencing in Fig. 4D.

Supplementary Material 4: Figure S4 *Igf2* and *H19* transcript levels in fetal liver. Total RNA was extracted from the livers of WT and *Kaiso* KO E12.5 embryos. *Igf2* (left) and *H19* (right) mRNA levels were measured by RT-qPCR. Each value represents the ratio of either *Igf2* or *H19* gene expression to that of *Gapdh*. The mean is indicated with horizontal bars.

Supplementary Material 5: Figure S5 DNA methylation status of the endogenous IG-DMR in *Kaiso* knockout mice. **A** Mouse *Dlk1*-*Dio3* locus structure. The enlarged map shows the IG-DMR and a black box indicates a repeat sequence. A gray bar below the map indicates the sequence analyzed by bisulfite pyrosequencing in (B) and (C). **B** and **C** DNA methylation status of endogenous mouse IG-DMR in tail somatic cells (B) and sperm (C) in the presence (WT) or absence (KO) of *Kaiso* was determined by bisulfite pyrosequencing.

## Data Availability

The datasets used and analyzed during the current study are available from the corresponding author on reasonable request.
