# Supplementary figures and images for: Imprinted DNA methylation of the H19 ICR is established and maintained in vivo in the absence of Kaiso
Source: Epigenetics Chromatin. 2024 Jun 5;17:20. doi: 10.1186/s13072-024-00544-8 (PMC11151560; doi:10.1186/s13072-024-00544-8)

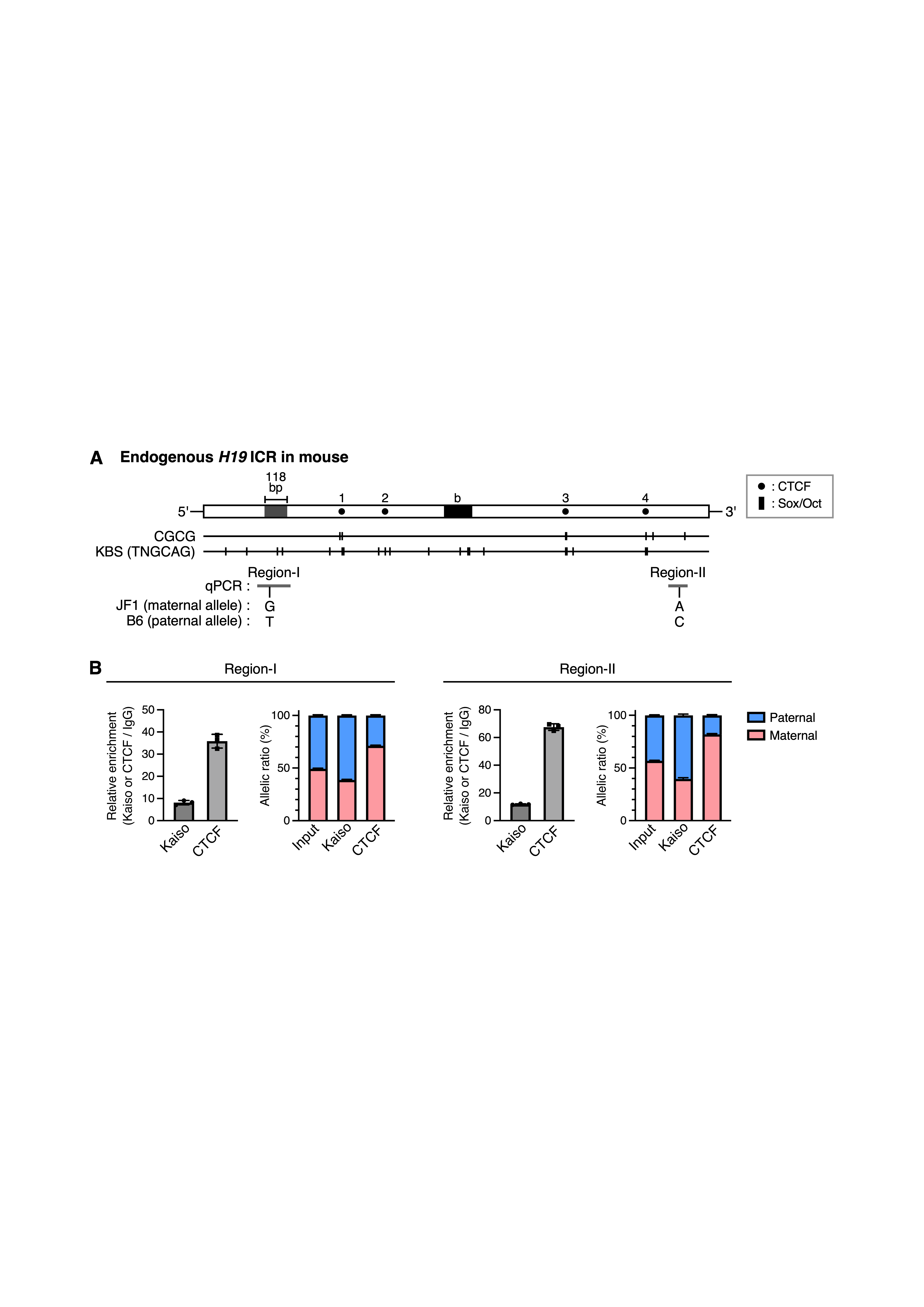

Supplement: Supplementary file 1 — Supplementary Material 1: Figure S1 Kaiso recruitment to endogenous mouse H19 ICR. A Mouse H19 ICR structure. Dots (1–4) indicate CTCF-binding sites. The black box indicates the 'b' region containing Sox-Oct motifs. Gray bars below the map indicate the regions amplified by qPCR (left, in each panel in B), which include SNPs analyzed by pyrosequencing (right, in each panel in B). B (Left in each panel) Chromatin from JF1/B6 hybrid mouse ES cells was immunoprecipitated using either control IgG, anti-Kaiso, or CTCF antibodies. Following qPCR analyses of the regions indicated in (A), relative enrichment values (Kaiso/IgG or CTCF/IgG signal ratios) were calculated. (Right in each panel) qPCR products were analyzed by pyrosequencing to determine binding allelic ratio using SNPs between JF1 (maternal allele) and B6 (paternal allele). Input chromatin was analyzed simultaneously as a control. CTCF bound more preferentially to the maternal allele than to the paternal allele, as previously reported [40, 41, 44]. [file 13072_2024_544_MOESM1_ESM.tif]

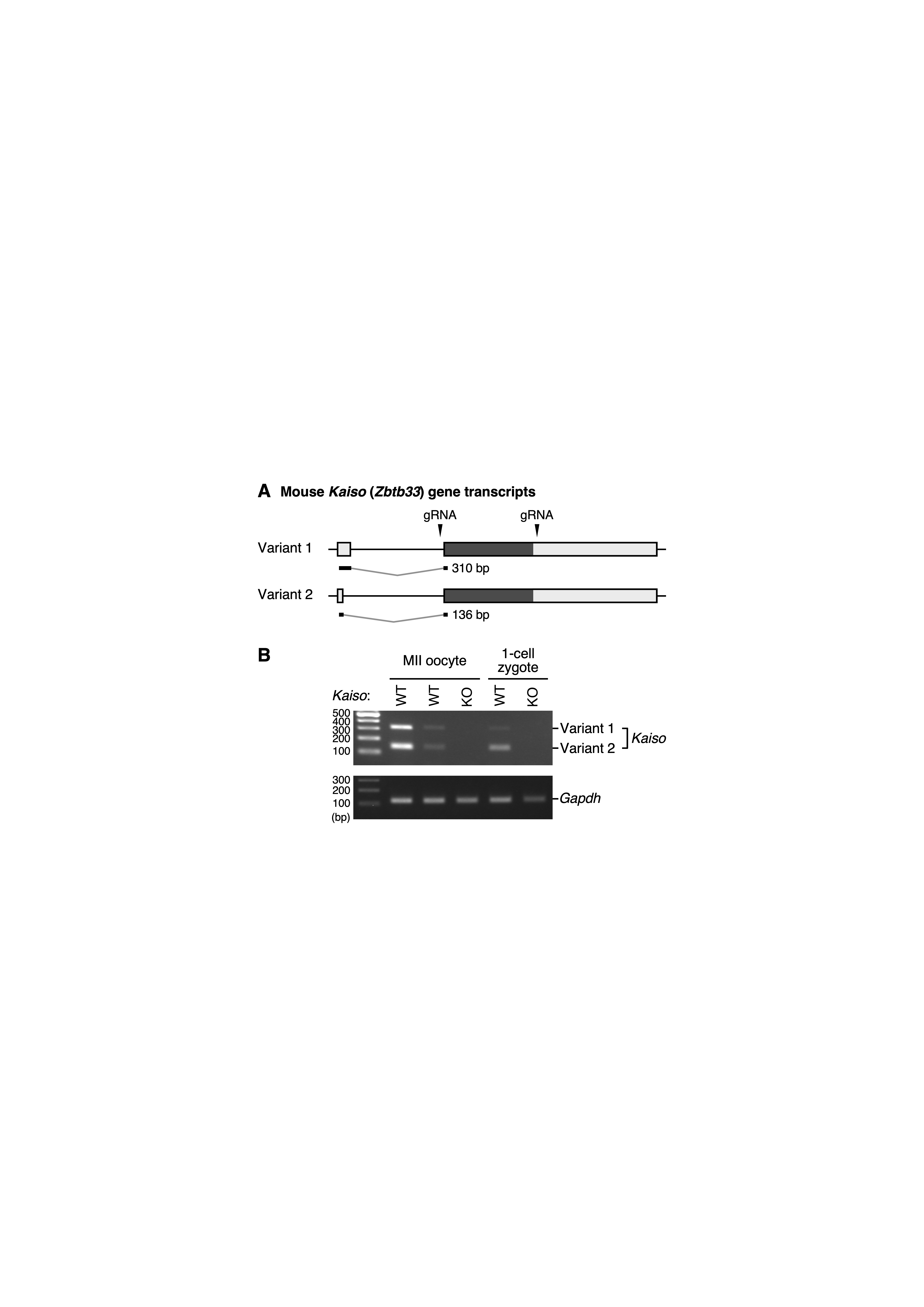

Supplement: Supplementary file 2 — Supplementary Material 2: Figure S2 Kaiso expression in oocytes and embryos. A Transcripts from Kaiso gene locus. Coding sequences in exons (rectangles) are indicated with filled boxes. Cas9 target sites for generating Kaiso gene knockout allele are indicated with arrowheads. cDNA regions amplified by PCR in (B) are indicated below the maps. B Total RNA was extracted from WT or Kaiso KO oocytes (30, 32, or 26 oocytes) and one-cell zygotes (31 or 12 embryos, respectively), reverse-transcribed, and the resulting cDNA was subjected to PCR using primer sets for either Kaiso or Gapdh. [file 13072_2024_544_MOESM2_ESM.tif]

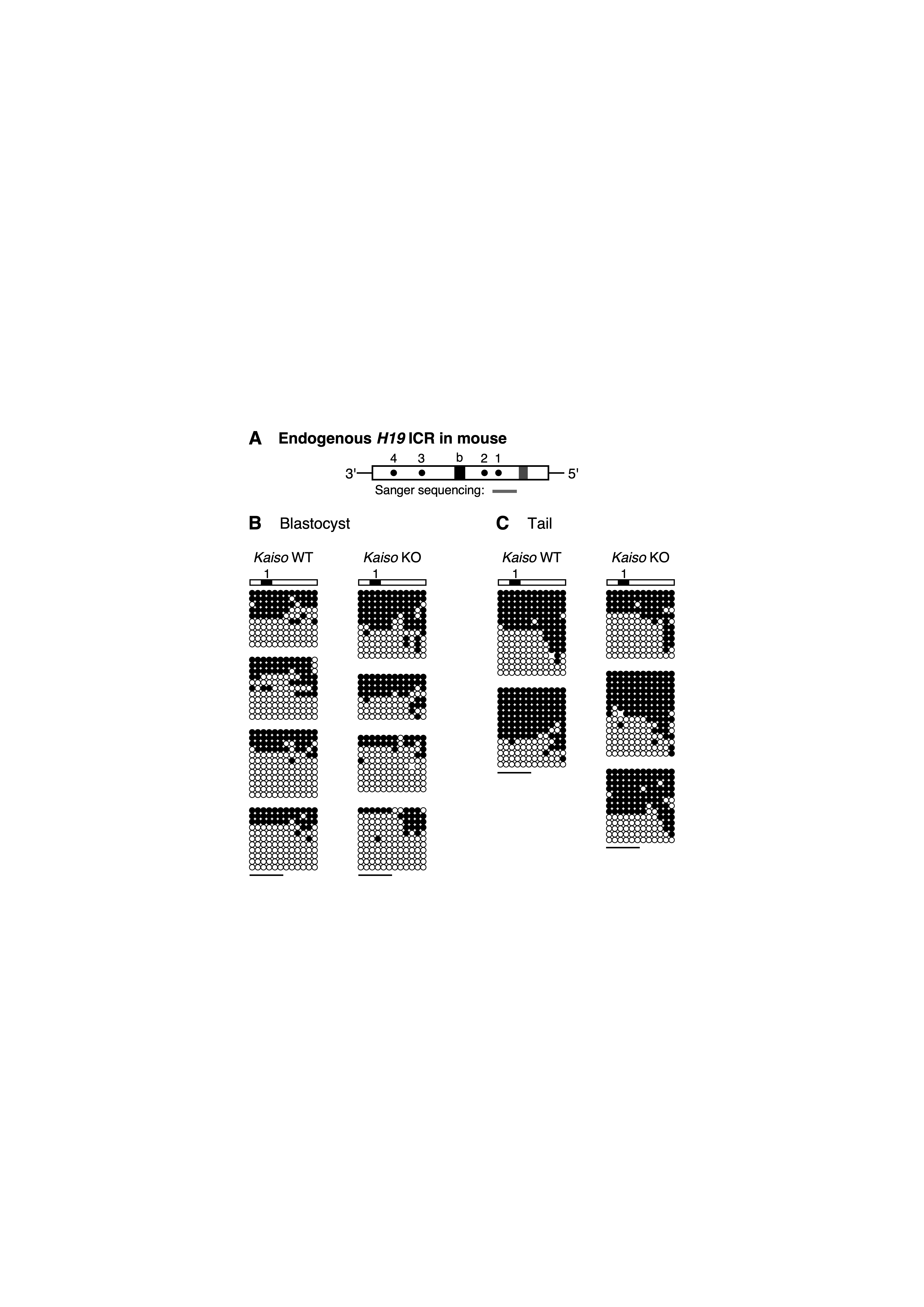

Supplement: Supplementary file 3 — Supplementary Material 3: Figure S3 DNA methylation status of endogenous H19 ICR in Kaiso knockout mice. A Map of the endogenous mouse H19 ICR fragment. The region analyzed by bisulfite Sanger sequencing in (B) and (C) is shown as a gray bar below the map. B DNA methylation status of H19 ICR in blastocysts in the presence (WT) or absence (KO) of Kaiso. Blastocyst stage embryos from a single litter were pooled and used as the sample. Each of four Kaiso WT or KO litters that were also analyzed by pyrosequencing in Fig. 4C were analyzed by Sanger sequencing. Bars under columns indicate CpGs analyzed by pyrosequencing in Fig. 4C. C DNA methylation status of H19 ICR in tail somatic cells from Kaiso WT or KO individuals that were also analyzed in Fig. 4D was determined by bisulfite Sanger sequencing. Bars under columns indicate CpGs analyzed by pyrosequencing in Fig. 4D. [file 13072_2024_544_MOESM3_ESM.tif]

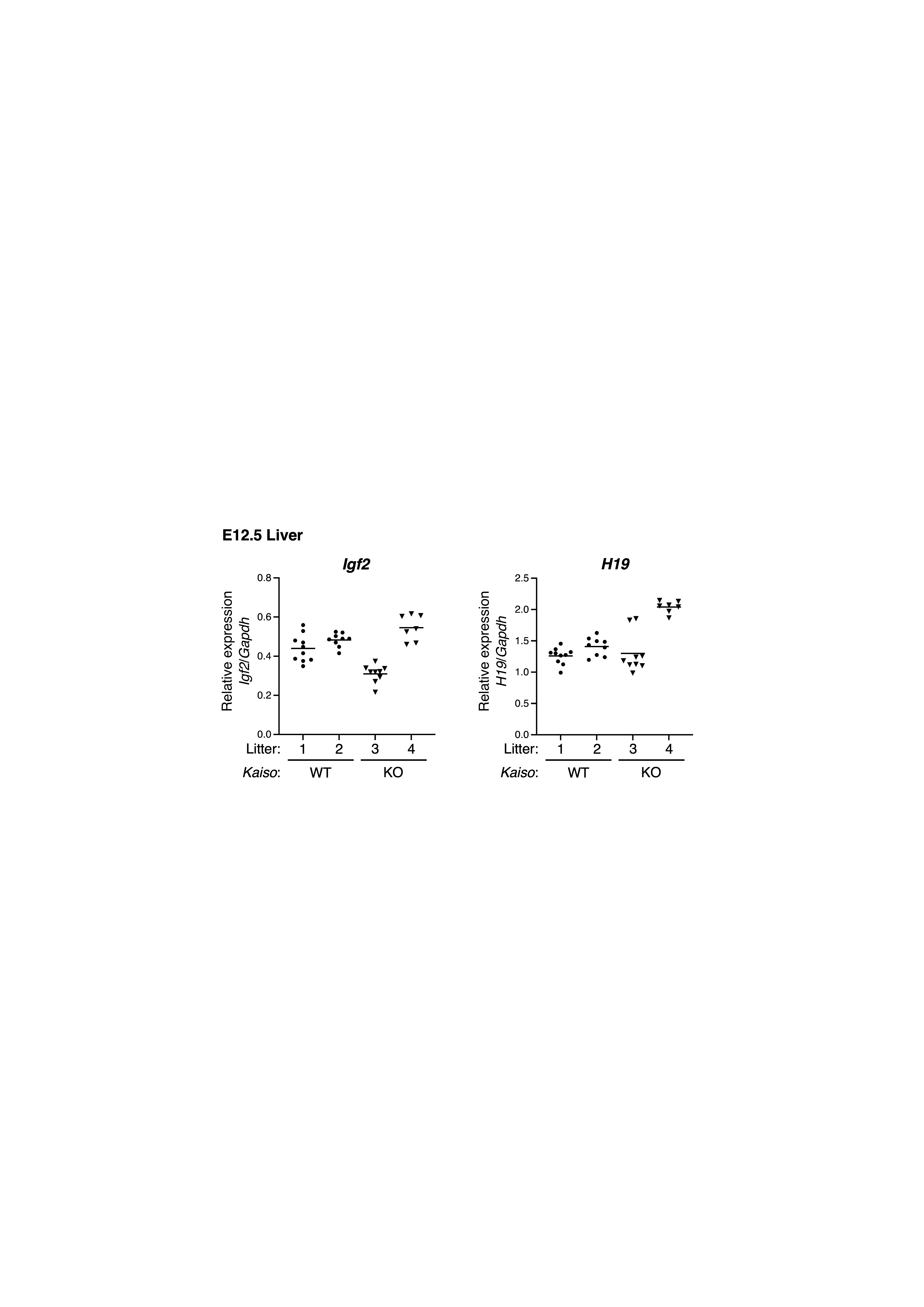

Supplement: Supplementary file 4 — Supplementary Material 4: Figure S4 Igf2 and H19 transcript levels in fetal liver. Total RNA was extracted from the livers of WT and Kaiso KO E12.5 embryos. Igf2 (left) and H19 (right) mRNA levels were measured by RT-qPCR. Each value represents the ratio of either Igf2 or H19 gene expression to that of Gapdh. The mean is indicated with horizontal bars. [file 13072_2024_544_MOESM4_ESM.tif]

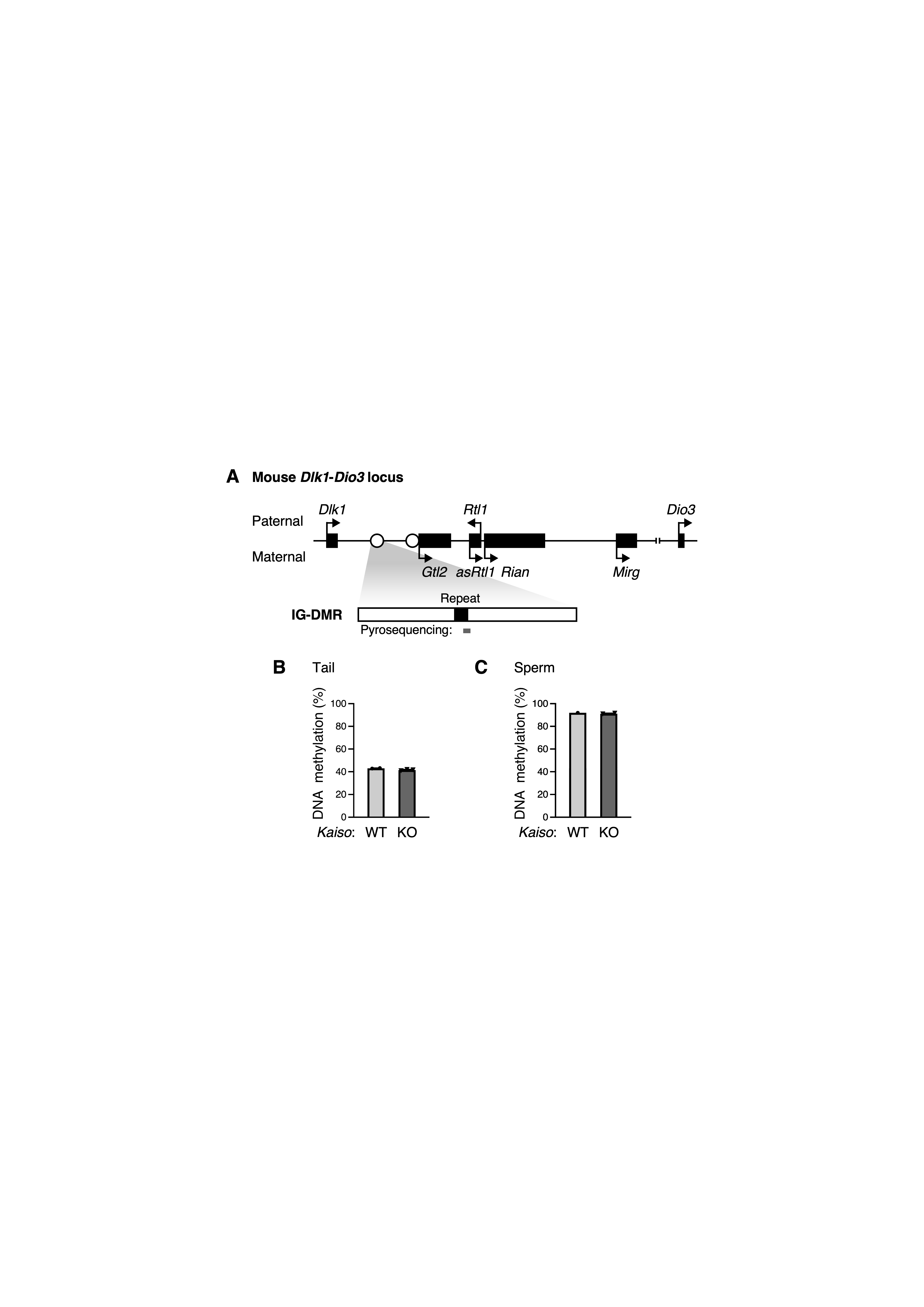

Supplement: Supplementary file 5 — Supplementary Material 5: Figure S5 DNA methylation status of the endogenous IG-DMR in Kaiso knockout mice. A Mouse Dlk1-Dio3 locus structure. The enlarged map shows the IG-DMR and a black box indicates a repeat sequence. A gray bar below the map indicates the sequence analyzed by bisulfite pyrosequencing in (B) and (C). B and C DNA methylation status of endogenous mouse IG-DMR in tail somatic cells (B) and sperm (C) in the presence (WT) or absence (KO) of Kaiso was determined by bisulfite pyrosequencing. [file 13072_2024_544_MOESM5_ESM.tif]
